# An Adaptive State- and Morphology-Based Four-Event Segmentation Algorithm for Sit-to-Stand-to-Sit Cycles from Wii Balance Board Load Signals: An Exploratory Comparison with Noraxon Report-Consistent IMU Timing

**DOI:** 10.3390/s26196267

**Published:** 2026-10-02

**Authors:** Xiaoliang Qiu, Yinghong Liu, Dae-Sung Park

**Affiliations:** Department of Physical Therapy, Konyang University, 158 Gwanjeodong-ro, Seo-gu, Daejeon 35365, Republic of Korea

**Keywords:** Wii Balance Board, sit-to-stand-to-sit, event segmentation, waveform morphology, load-state estimation, inertial measurement unit, phase-specific analysis

## Abstract

Sit-to-stand (STS) tests are often summarized by completion time or repetition count; however, phase-specific analysis requires reproducible operational segmentation. We developed an adaptive state- and morphology-based algorithm that identified ten internal anchors in Wii Balance Board (WBB) vertical-load waveforms and derived an ordered P1–P4 chain representing cycle start, end of sit-to-stand, start of stand-to-sit, and cycle end, respectively. The algorithm was developed using 108 T1–T3 trials and then applied unchanged to a temporally separate batch of 24 T0 trials. Fourteen T0 participants overlapped with development and 10 were included in a descriptive subset of participants not represented in the development dataset. Complete chains were identified in 24/24 T0 trials and 10/10 descriptive-subset trials (100%; exact 95% CI, 69.2%–100%), versus 7/24 for the prespecified internal non-adaptive baseline. Complete-chain identification remained 100% across all three datasets under one-at-a-time perturbations of ±10% and ±20% of 52 frozen numerical parameters, although selected sit-down rules shifted local boundaries. Removing the separate seated-baseline/fallback component reduced complete-chain identification to 101/108, 12/24, and 8/10. Across 131 paired trials, WBB total-cycle duration differed from Noraxon software (MR 4.2.34 Software; Noraxon, Scottsdale, AZ, USA)-reported duration by −0.188 s (95% CI, −0.313 to −0.060). These differences reflect distinct operational timing definitions rather than error against an independent reference standard. Independent reference-event and external clinical studies remain necessary.

## 1. Introduction

Sit-to-stand (STS) performance is clinically informative because it reflects the integrated ability to generate, transfer, and control body load during rising and sitting. Completion time and repetition count remain useful global outcomes; however, they do not identify when loading, standing stabilization, or return-to-sit transitions occur. Phase-specific timing and waveform morphology can, therefore, add process-level information for movement assessment and sensor-based monitoring. The five-times sit-to-stand test is a conventional functional outcome with established test–retest reliability [1], while instrumented STS testing provides phase-specific information beyond manually recorded timing [2].

Instrumented STS studies illustrate complementary ways to obtain process-level information. Greene et al. [3] examined wearable-sensor features for balance, cognition, and estimation of fall risk during STS, while Wairagkar et al. [4] modeled STS kinematics from inertial sensors. Hosseini and Ghahramani [5] used an instrumented five-times STS test with machine learning to assess locomotive syndrome. Millor et al. [6] synthesized kinematic parameters for sit-to-stand and stand-to-sit assessment. Hellec et al. [7] evaluated an IMU embedded in smart glasses against an optoelectronic system, providing an example of an independent reference-based validation design. More recently, Chan et al. [8] used smartphone-derived joint angular velocities and a deep learning model to characterize symptomatic knee osteoarthritis, demonstrating the clinical potential of low-cost video-derived STS dynamics. 

These studies differ in sensor modality, target outcome, and reference design; their operational boundaries should therefore not be assumed to be interchangeable.

The Wii Balance Board (WBB) offers a low-cost mechanical-load channel that can complement kinematic sensors. Prior WBB work has addressed standing-balance validity, center-of-pressure measurement, transitional loading, weight-bearing asymmetry, and STS performance [9,10,11,12,13,14,15,16,17]. However, most of these studies do not provide a transparent adaptive procedure for segmenting a complete STS cycle into multiple operational boundaries. Natural WBB waveforms may contain preparatory unloading, false start-like excursions, short standing intervals, competing peak–trough candidates, unequal seated baselines, and delayed post-sitting stabilization. A single fixed threshold may, therefore, be vulnerable to waveform-morphology variability.

This study addresses that signal-processing problem with an adaptive state- and morphology-based procedure. The algorithm constructs an ordered ten-anchor chain, scores complete candidates, and applies prespecified conditional recovery and boundary-refinement rules. The prespecified internal non-adaptive baseline is used only as a within-dataset comparator, not as a proxy for the published segmentation landscape. The WBB–Noraxon analyses compare operational timing definitions derived from mechanical-load and kinematic signals; because independent sample-level reference events were unavailable, they are not a criterion-standard validation of event-detection accuracy.

The primary objective was to develop the transparent adaptive algorithm and evaluate complete ordered P1–P4 chain identification relative to the prespecified internal non-adaptive baseline. The second objective was to compare WBB-derived total-cycle duration with Noraxon software-reported duration as an operational timing comparison. We also examined synchronized boundary differences descriptively. Additional post hoc analyses characterized numerical and architectural robustness using the frozen sensitivity and ablation procedures.

## 2. Materials and Methods

### 2.1. Study Design and Participants

This algorithm-development study comprised three linked analyses: development and internal evaluation of WBB event segmentation, paired comparison of WBB and Noraxon total-cycle timing, and exploratory comparison of synchronized cycle boundaries. The two acquisition batches were assigned different analytic roles rather than being created by randomly splitting one dataset. The later T1–T3 batch comprised three trials from each of 36 participants (108 trials) and was the sole dataset used to define the WBB event logic, numerical parameters, prespecified internal non-adaptive baseline, and final completeness and plausibility criteria. The earlier T0 batch comprised one trial from each of 24 participants. Although T0 was collected approximately one month before T1–T3, an early technical T0 test artifact existed before the final formal evaluation but was used only to verify file import and pipeline execution; it was not used to modify algorithm rules, numerical parameters, quality-control criteria, or comparator selection. Final Adaptive v9 was stabilized and frozen using the 108 T1–T3 development trials and then rerun unchanged on T0 for formal evaluation.

Fourteen participants contributed records to both acquisition batches; therefore, the 36 development participants and 24 internal-evaluation participants represented 46 unique individuals. The internal-evaluation batch was not fully participant-independent and is, therefore, treated as a temporally separate internal evaluation batch rather than an independent external-validation cohort. Formal participant crosswalk records confirmed that the remaining 10 T0 participants were not represented in the 108-trial development dataset; these 10 trials were retained as a small participant-independent descriptive subset. The subgroup was not considered an adequately powered independent validation cohort.

Participants were eligible if they were 18–60 years of age, could understand and follow the test instructions, could complete one sit-to-stand-to-sit cycle independently and safely without an assistive device, and had body mass within the safe operating range of the WBB. Exclusion criteria were acute pain during testing, a musculoskeletal or neurological condition affecting task performance, inability to complete the task safely, or any condition judged by the investigator to make testing inappropriate. The final sample comprised 23 women and 23 men; mean age was 26.8 ± 6.2 years, mean height was 1.68 ± 0.08 m, mean body mass was 68.2 ± 14.5 kg, and mean body mass index was 24.1 ± 4.3 kg/m^2^.

The study was approved by the Konyang University Institutional Review Board (approval no. KYU 2026-04-022-001; date of approval: 22 May 2026); all participants provided written informed consent before data collection. The participant characteristics and dataset structure are summarized in Table 1.

Values are mean ± SD unless otherwise stated. Fourteen participants contributed to both acquisition batches; therefore, the two batches represented 46 unique participants. Ten participants in the internal-evaluation batch were not represented in the development batch. BMI, body mass index.

### 2.2. Experimental Procedure

**Figure 1:**
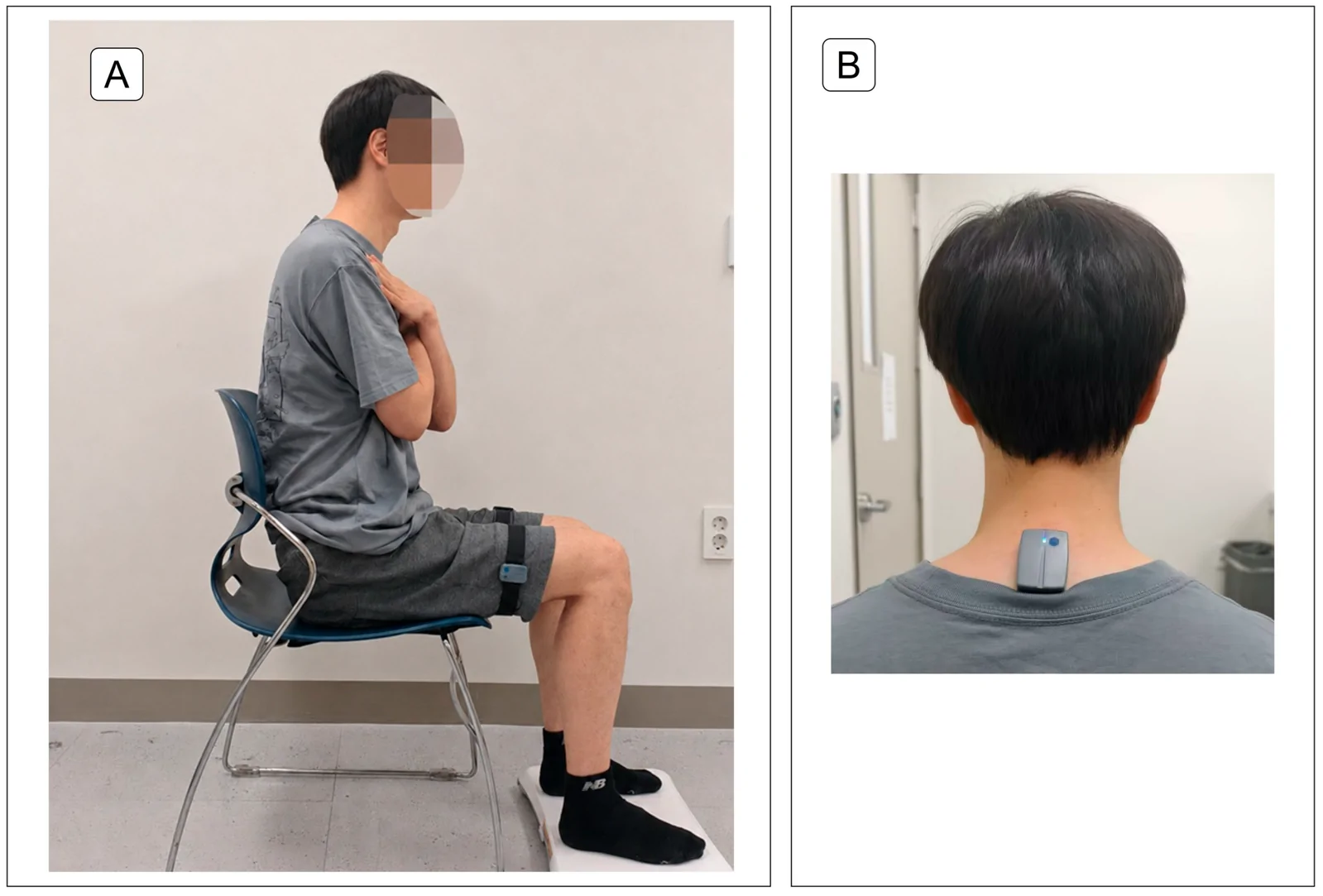
Experimental setup and inertial-sensor placement: (**A**) initial seated position on a 0.45 m chair with both feet on the Wii Balance Board and the arms crossed over the chest; the right-thigh Ultium Motion inertial measurement unit is visible. Foot placement was self-selected, and inter-foot distance was recorded; (**B**) posterior placement of an identical Ultium Motion inertial measurement unit over the C7 region.

Participants wore socks and sat on a 0.45 m chair with both feet positioned on the WBB and the arms crossed over the chest. Foot distance was not standardized to a fixed value; each participant selected a comfortable position that permitted a natural sit-to-stand-to-sit movement and the inter-foot distance was recorded. The standardized instruction was: “Please stand up naturally to a fully upright position and then sit down once.” No external assistance was provided. The initial seated posture is shown in Figure 1A.

Each recording lasted approximately 40 s; repeated trials were separated by approximately 20–25 s. One complete natural-speed sit-to-stand-to-sit cycle was performed in each trial. The development batch contained three repeated trials per participant, whereas the internal evaluation batch contained one trial per participant. Approximately 5 s before the movement in the repeated development recordings, participants performed one seated right-foot stomp. The stomp was used only for WBB–Noraxon time-axis alignment and was excluded from WBB baseline estimation, dynamic-amplitude estimation, peak detection, and sit-to-stand-to-sit event identification.

### 2.3. Instrumentation

WBB data were recorded using a Nintendo Wii Balance Board (model RVL-021; Nintendo Co., Ltd., Kyoto, Japan) and Balancia 3.0 software (developed by Dae-Sung Park, Konyang University, Daejeon, Republic of Korea) and exported as time-stamped vertical-load values in kilograms together with center-of-pressure records. The exported files contained trial-specific time stamps. The effective sampling frequency calculated from adjacent time stamps was approximately 100 Hz and all time-based algorithm windows were converted to trial-specific sample counts. Because laboratory force-platform calibration in Newtons was not established for this analysis, the primary signal is described as vertical load rather than laboratory-grade ground-reaction force. Previous work has evaluated WBB validity and reliability for standing balance, center-of-pressure measurement, and transitional tasks [9,10,11,12,17].

Noraxon inertial data were recorded simultaneously using an Ultium Motion system (Noraxon U.S.A., Inc., Scottsdale, AZ, USA) at a configured sampling frequency of 200 Hz and processed using MR software, version 4.2.34. Identical Ultium Motion inertial measurement units were positioned over the posterior C7 region and on both thighs. The C7-mounted IMU provided forward-lean angle and sagittal angular-velocity signals for identifying the kinematic phase-separation anchor, whereas the right-thigh-mounted IMU provided the kinematic impact landmark used for seated-stomp synchronization. Posterior C7 placement is shown in Figure 1B. Noraxon software-generated phase and cycle durations were treated as software-reported summary outcomes rather than sample-level reference event labels.

### 2.4. WBB Signal Preprocessing, Movement-Interval Localization, and Load-State Estimation

**Figure 2:**
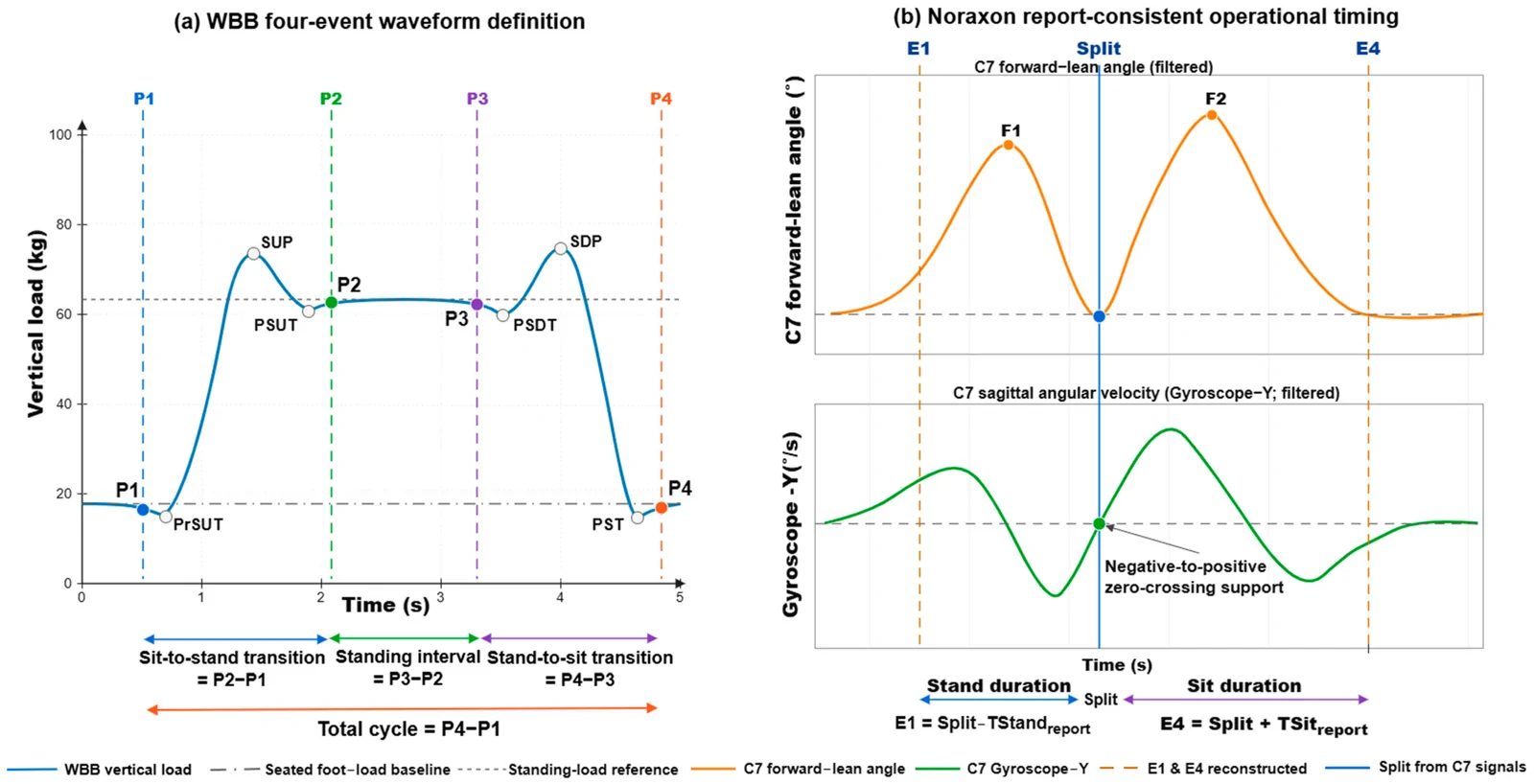
Operational timing definitions for WBB and Noraxon: (**a**) WBB four-event definition: P1, cycle start and first sustained departure from the seated foot-load baseline; P2, return to the standing-load reference after the standing-up peak and trough; P3, departure from the standing-load state before the pre-sitting trough; and P4, stable return to the post-sitting seated baseline. Derived intervals are sit-to-stand (P2 − P1), standing (P3 − P2), stand-to-sit (P4 − P3), and total cycle (P4 − P1); and (**b**) Noraxon report-consistent operational timing: Split is the local minimum between the two principal filtered C7 forward-lean peaks, supported by a negative-to-positive zero crossing of filtered C7 sagittal angular velocity (Gyroscope-Y); E1 and E4 are reconstructed as Split − TStand_report_ and Split + TSit_report_, respectively. WBB and Noraxon boundaries are operational definitions from different signal domains. The waveforms are schematic; amplitudes are not intended for quantitative comparison.

The adaptive method reported four principal boundaries: P1, cycle start; P2, end of the sit-to-stand transition; P3, start of the stand-to-sit transition; and P4, cycle end. Corresponding durations were sit-to-stand (P2 − P1); standing interval (P3 − P2); stand-to-sit (P4 − P3); and total cycle (P4 − P1). When the standing interval could not be separated within one effective sample and the prespecified merge criteria were satisfied, P2 and P3 were allowed to coincide, and standing-interval duration was recorded as zero (Figure 2a).

All numerical settings were established using only the 108 T1–T3 development trials and were fixed before formal T0 evaluation. After invalid observations were removed, duplicate time stamps were averaged, and samples were ordered by time. Trial-specific sampling frequency was estimated from adjacent time stamps and all time-based windows were converted to trial-specific sample counts. The WBB vertical-load signal was processed using a centered 0.05 s rolling median followed by a centered 0.075 s moving mean. These empirical engineering settings were established during waveform review and rule stabilization in the development set rather than through grid search or statistical optimization. At an effective sampling frequency of approximately 100 Hz, the windows corresponded to about 5 and 7–9 samples, respectively. The median window suppressed isolated spikes, whereas the subsequent moving mean provided light smoothing for derivative and local-extrema calculations, while limiting temporal averaging across major load transitions.

The principal sit-to-stand-to-sit movement was localized by identifying the dominant sustained excursion above a trial-adaptive activity threshold. The search interval was extended before and after this excursion to include the complete movement while excluding the earlier seated-stomp synchronization signal.

Stable pre- and post-movement seated periods were used to estimate the seated-load state. Compatible periods were combined; separate pre- and post-movement estimates were used only when prespecified endpoint or event-sequence conditions indicated that a common seated state was unsuitable. Standing-load references were subsequently derived from the high-load region between the standing-up and sitting-down transitions. Complete preprocessing, window, threshold, and fallback specifications are provided in Supplementary Table S4.

### 2.5. Adaptive State- and Morphology-Based Ordered-Event-Chain Segmentation, Boundary Refinement, and Internal Evaluation

Rather than detecting P1–P4 as four independent threshold crossings, the adaptive algorithm identified them within an ordered ten-anchor event chain:

P1 < PrSUT < SUP < PSUT < P2 ≤ P3 < PSDT < SDP < PST < P4

Here, PrSUT, SUP, and PSUT denote the pre-standing-up trough, standing-up loading peak, and post-standing-up trough, respectively; PSDT, SDP, and PST denote the pre-sitting-down trough, sitting-down loading peak, and post-sitting trough, respectively. P1 marked the final sustained departure from the pre-movement seated state, P2 and P3 described entry into and exit from the standing-load state, and P4 marked stable return to the post-movement seated state.

The algorithm first assembled a provisional standard chain. When prespecified endpoint, standing-state, or sequence conditions were not satisfied, alternative candidates could be generated using separate seated baselines, recovered standing episodes, or phase-specific peak–trough morphology. Candidates violating the required event order, transition-duration requirements, endpoint criteria, or total-cycle plausibility were discarded.

**Figure 3:**
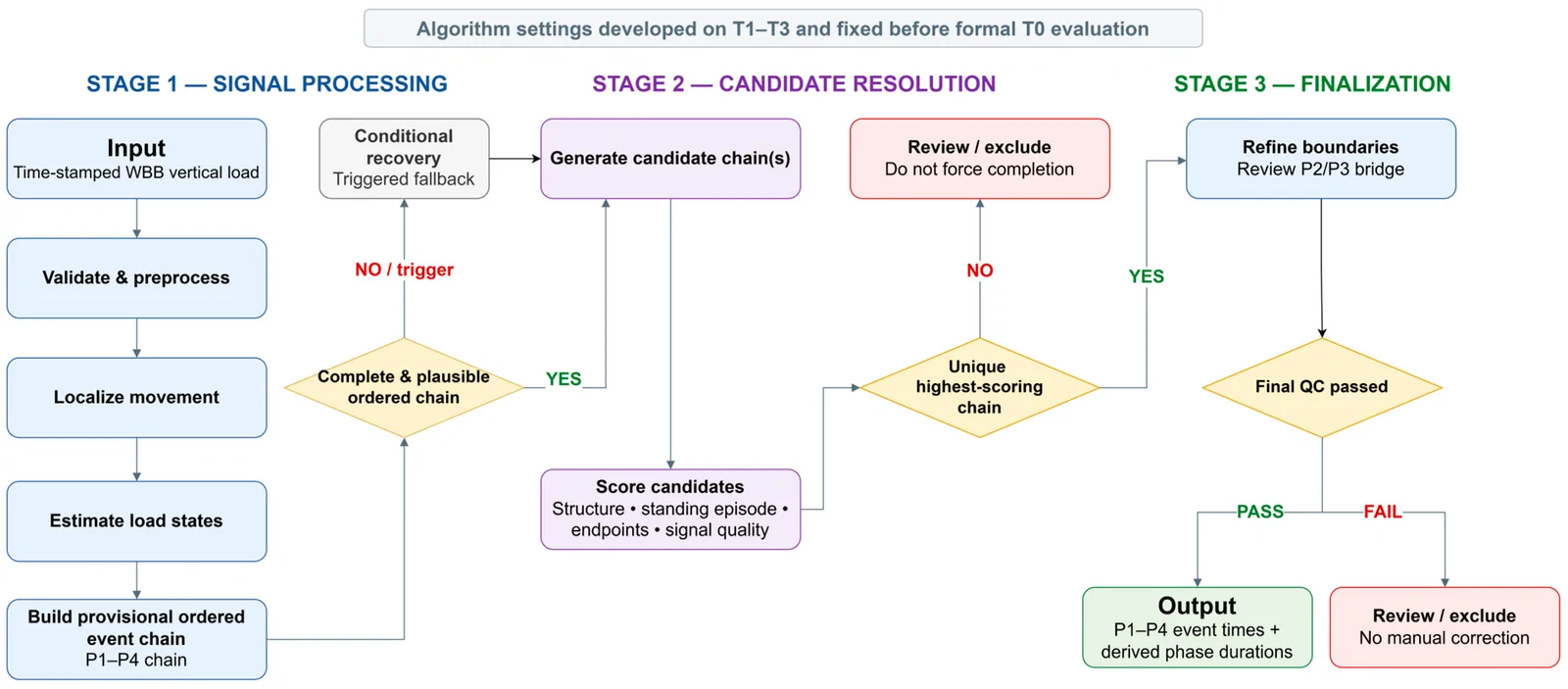
Control flow of the adaptive state- and morphology-based four-event segmentation algorithm. All settings were developed on the 108 T1–T3 development trials and fixed before formal evaluation on the 24 T0 trials. Following preprocessing, movement localization, load-state estimation, and construction of a provisional ordered P1–P4 chain, prespecified recovery is triggered only when completeness or plausibility criteria are not met. Candidate chains are then generated and scored, while unresolved ambiguity is classified as Review or Exclude without forced completion. The selected chain undergoes boundary refinement and final quality control before event times and phase durations are output. Colors indicate functional roles: blue, primary processing and boundary refinement; purple, candidate generation and scoring; yellow, decisions; green, successful completion and output; red, review or exclusion; and gray, auxiliary or conditional information. Detailed criteria are provided in Supplementary Tables S4 and S5.

The remaining complete chains were scored using peak–trough structure, standing-state quality, P2/P3 transition evidence, endpoint plausibility, duration plausibility, and signal-quality evidence. Unresolved candidate ambiguity resulted in a Review classification rather than manual selection. For the selected chain, P2 and P3 were re-evaluated against the dominant continuous standing-load segment, and P1, P3, or P4 underwent additional refinement only when the prespecified morphology conditions were met. Detailed candidate-generation, scoring, ambiguity, and boundary-refinement rules are provided in Supplementary Table S5 and are summarized in Figure 3.

The finalized algorithm was applied without modification to the 24 T0 internal-evaluation trials. An OK result required a complete ordered chain, nonnegative phase durations, a total-cycle duration of 2–8 s, and no unresolved endpoint, re-entry, or candidate-ambiguity condition. Otherwise, a Review or Exclude classification and the corresponding reason were recorded without forcing boundary completion.

Because 14 T0 participants also contributed T1–T3 development trials, the full T0 batch was interpreted as a temporally separate internal evaluation batch with participant overlap rather than an independent external-validation cohort. The remaining 10 T0 participants formed a participant-independent descriptive subset and were examined separately.

**Figure 4:**
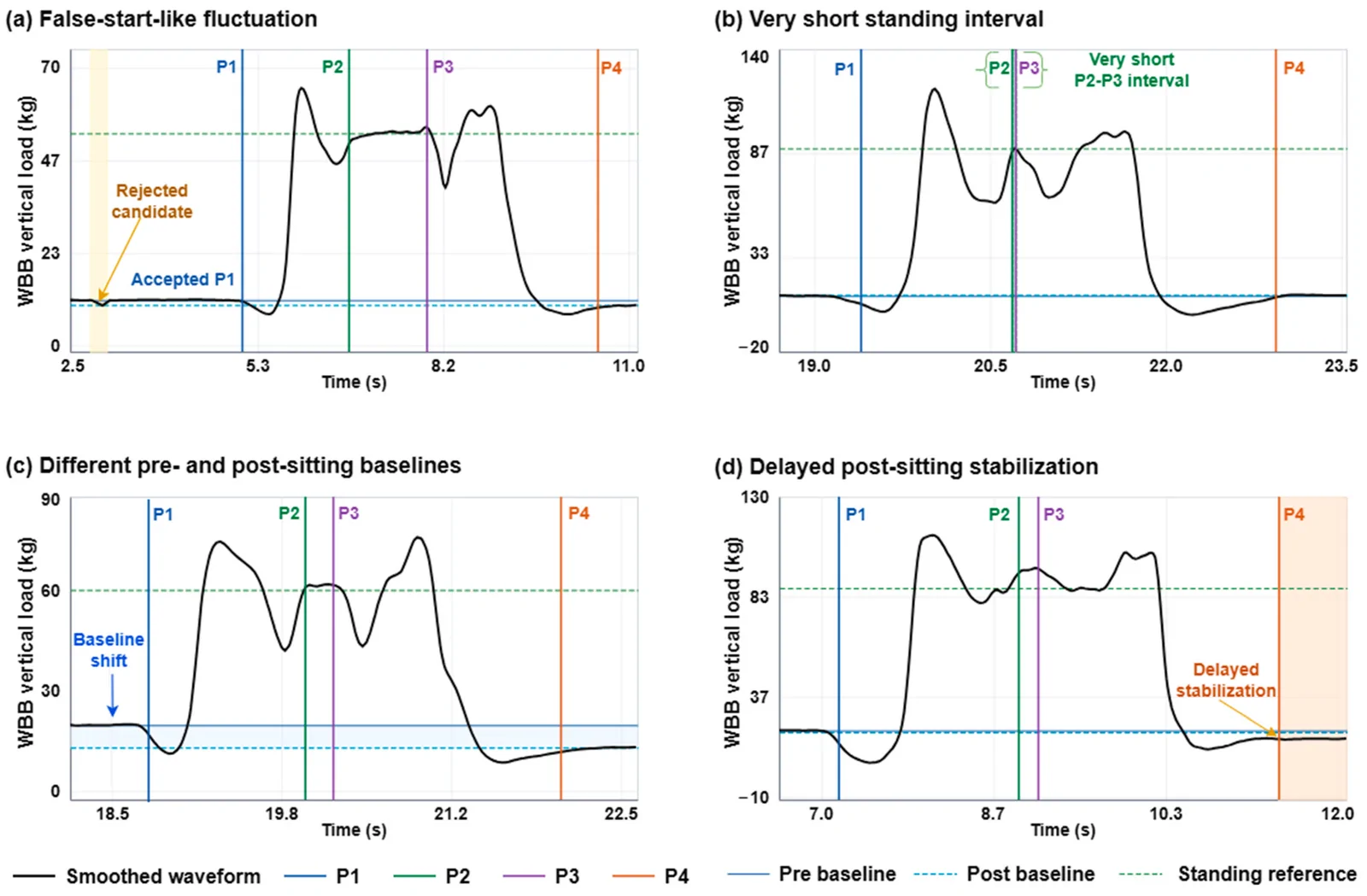
Representative WBB vertical-load waveforms illustrating adaptive morphology handling: (**a**) rejection of a false-start-like early candidate and retention of a later sustained P1; (**b**) a very short P2 − P3 standing interval; (**c**) different pre- and post-sitting baselines; (**d**) delayed post-sitting stabilization affecting P4. In panel (**d**), the light-orange shaded area highlights the post-P4 interval, illustrating the delayed stabilization of the WBB signal. Black curves show the smoothed load signal, colored vertical lines indicate P1 − P4, and horizontal lines indicate the pre- and post-sitting baselines and standing reference. The axes show time (s) and vertical load (kg). These panels illustrate algorithmic decision conditions and are not independent reference-event annotations.

Structured waveform review was used only to document interpretability and quality-control status. It did not alter P1–P4 times or trigger post hoc changes to the frozen algorithm. Representative waveform conditions, including false starts, short standing intervals, baseline differences, and delayed stabilization, are illustrated in Figure 4.

### 2.6. Prespecified Internal Non-Adaptive Baseline

A deterministic fixed-threshold method (FixedThreshold_D_v1.3_FROZEN) was specified using the T1–T3 development dataset and frozen before formal processing of T0. This method served as a prespecified internal non-adaptive baseline rather than as a representative published segmentation approach. Its purpose was to characterize the difference in the rate of complete ordered P1–P4 chain identification between a simplified non-adaptive implementation and the adaptive architecture using the same WBB vertical-load signal. The baseline did not include candidate-chain scoring, adaptive standing-state updating, morphology-based recovery procedures, external-device information, trial-specific hard coding, or manual event-time correction. Trials without a complete ordered chain remained in the denominator. Direct superiority over published rule-based, template-based, or machine-learning segmentation methods was not tested.

The prespecified internal non-adaptive baseline used a centered 0.05 s moving average, stable 1.0 s pre- and post-sitting baseline windows, dynamic amplitude defined from the 99th percentile, fixed height and prominence requirements for the two principal peaks, and an 80th-percentile standing-load reference. P1–P4 were derived from fixed baseline- or standing-state crossings with prespecified confirmation and occupancy requirements. Trials without a complete strictly ordered chain remained in the analysis denominator. The full frozen parameter and failure-rule specification is provided in Supplementary Table S6. This baseline was not intended to represent all published sit-to-stand segmentation approaches.

### 2.7. Noraxon Report-Consistent Kinematic Boundary Reconstruction

Noraxon software provided standing and sitting phase durations but did not export corresponding sample-level event labels. For the 107 development trials with valid same-trial Noraxon records, report-consistent kinematic boundaries were, therefore, reconstructed from the C7 forward-lean angle and sagittal angular velocity.

The phase-separation point, Split, was identified as the local minimum of the angle signal between the two principal forward-lean peaks, supported by a negative-to-positive sagittal angular-velocity zero crossing. Report-consistent E1 and E4 locations were defined relative to this signal-supported Split using the software-reported Stand and Sit durations:

E1 = Split − Stand Duration;

E4 = Split + Sit Duration

Each outer boundary was additionally classified as signal-supported, report-constrained, or duration-derived according to the degree and route of outer-boundary signal support. These categories described provenance rather than three different outcome definitions or independent accuracy grades. Because independent sample-level Noraxon event labels were unavailable, the cross-device analysis was interpreted as a comparison of operational timing definitions, not criterion-standard validation. Detailed reconstruction criteria and provenance definitions are provided in Supplementary Figure S1, Supplementary Table S1A–C, and Supplementary Note S1.

### 2.8. Seated-Stomp Synchronization and Boundary Differences

For repeated development trials, seated-stomp impact landmarks were detected independently in the WBB and Noraxon signals and paired using frozen cross-system criteria. The primary synchronization offset was defined as WBB impact-onset time minus Noraxon impact-onset time. WBB event times were mapped to the Noraxon time axis by subtracting this offset.

A synchronization value was accepted only when a unique candidate pair satisfied the prespecified morphology and cross-system consistency checks; no synchronization value was imputed. Of 108 planned repeated development trials, 107 had valid paired WBB and Noraxon records. Fourteen Noraxon records lacked a detectable seated stomp, leaving 93 synchronization-eligible trials. Reliable synchronization was achieved in 75 trials; the other 18 were excluded from boundary-difference analysis, with reasons retained in the audit record. Detailed synchronization and quality-control criteria are provided in Supplementary Note S1.

After synchronization, boundary differences were defined as:

ΔStart = WBB P1 − Noraxon E1; ΔEnd = WBB P4 − Noraxon E4; ΔDuration = WBB total duration − Noraxon total duration.

Negative values indicated an earlier WBB boundary or shorter WBB duration. The identity ΔDuration = ΔEnd − ΔStart was used to determine whether start and end shifts were partially cancelled or accumulated.

### 2.9. Analysis Sets and Outcomes

The WBB algorithm dataset comprised all 132 trials. The primary internal evaluation included the 24 T0 trials. The descriptive participant-independent check comprised the 10 T0 participants who had not contributed to T1–T3 development.

The total-cycle comparison included 131 trials with both WBB-derived and Noraxon software-reported durations. Noraxon boundary reconstruction included 107 development trials. Synchronization was evaluable in 93 trials, and exploratory synchronized boundary analysis included the 75 trials with reliable synchronization and complete WBB P1/P4 and Noraxon E1/E4 boundaries.

The primary internal-evaluation outcome was whether a complete ordered P1–P4 chain was identified. This outcome was compared between the adaptive algorithm and the prespecified internal non-adaptive baseline, while reasons for incomplete baseline identification were summarized separately. Cross-device outcomes were total-cycle difference and limits of agreement. Exploratory synchronized outcomes were ΔStart, ΔEnd, and ΔDuration. Timing comparisons restricted to trials in which both implementations produced a complete sequence were considered conditional supportive analyses.

### 2.10. Statistical Analysis

Continuous variables were summarized using means and standard deviations or medians and interquartile ranges, as appropriate. Within the 24 T0 trials, paired complete P1–P4 identification was compared between methods using the exact McNemar test. The paired absolute difference and its exact 95% confidence interval were also reported. Results for the 10 participant-independent T0 trials were summarized separately, with exact confidence intervals as a descriptive participant-independent check.

Among trials successfully segmented by both methods, paired absolute WBB-minus-Noraxon total-cycle differences were compared using the Wilcoxon signed-rank test. This conditional analysis was interpreted as supportive and not as an assessment of criterion accuracy.

For the 131-trial cross-device comparison, differences were defined as WBB minus Noraxon. Mean difference, mean absolute difference, root-mean-square difference, and descriptive trial-level Bland–Altman limits were calculated [18]. Because participants contributed repeated trials, participant-cluster bootstrap confidence intervals and a random-intercept variance-component sensitivity analysis were used [19]. Cluster-robust models assessed whether the mean difference differed from zero, proportional bias, and differences in mean bias between the internal-evaluation and development batches. Participant-mean ICC(A,1) was reported as a supportive agreement descriptor rather than as a single-trial ICC [20].

For the 75 synchronized trials, participant-cluster bootstrap confidence intervals were calculated for ΔStart, ΔEnd, and ΔDuration. Pearson correlation described the association between ΔStart and ΔEnd but was interpreted descriptively because the two measures shared reconstruction and synchronization components. Selection into the synchronized analysis set was examined in sensitivity analyses, with detailed methods and results provided in Supplementary Table S2A–C.

No missing outcomes were imputed. Tests were two-sided with *α* = 0.05. The verified formal-analysis rerun environment used Python 3.13.5, NumPy 2.3.5, SciPy 1.17.0, statsmodels 0.14.6, pandas 2.2.3, and Matplotlib 3.10.8, with a fixed random seed. The frozen algorithm, statistical scripts, environment specifications, and reproducibility manifests were retained for versioned release or controlled inspection.

### 2.11. Parameter Sensitivity and Targeted Component Ablation

To examine whether complete-chain performance depended on narrowly tuned numerical settings, an additional post hoc parameter-sensitivity analysis was conducted. The parameter list, perturbation grid, and outcome definitions were fixed before sensitivity outcomes were generated. Fifty-two frozen numerical parameters were perturbed, one at a time, to 80%, 90%, 100%, 110%, and 120% of their frozen values, while all other numerical settings and algorithmic logic remained unchanged. Analyses were performed in the 108-trial development dataset, the full 24-trial T0 batch, and the 10-trial participant-independent descriptive subset. Outcomes included complete ordered P1–P4 chain identification, absolute P1–P4 boundary shifts relative to the frozen implementation, total-cycle duration shifts, and the location of any chain failure.

A targeted component-ablation analysis was also performed to characterize the contribution of major algorithmic modules. Each prespecified module was disabled individually without re-optimizing the remaining parameters, with the full frozen Adaptive v9 implementation serving as the reference. The exact frozen module identifiers are reported in Supplementary Table S8. These analyses were intended to characterize numerical and architectural robustness and were not interpreted as assessments of event-detection accuracy.

### 2.12. Use of Generative Artificial Intelligence

OpenAI Codex (cloud service; accessed in 2026) was used to assist with source-code and reproducibility-package preparation, statistical and document quality-control workflows, and manuscript language and formatting. The authors independently reviewed and verified the code, numerical results, figures, tables, and text used in this study.

## 3. Results

### 3.1. Participant and Trial Flow

The study included 46 participants and 132 WBB trials. A complete ordered four-event sequence was identified in all 132 WBB trials, and 131 had a paired Noraxon total-cycle result. Of 107 development trials available for Noraxon boundary reconstruction, 93 contained a usable seated stomp in both systems and 75 were reliably synchronized for the exploratory boundary analysis.

### 3.2. Internal Evaluation Against the Prespecified Internal Non-Adaptive Baseline

In the 24-trial internal-evaluation batch, the adaptive algorithm generated a complete ordered P1–P4 sequence in 24/24 trials (100.0%), compared with 7/24 trials (29.2%) for the prespecified internal non-adaptive baseline (paired difference, 70.8 percentage points; 95% CI, 48.9–87.4; exact McNemar *p* < 0.001). This comparison concerns algorithmic complete-chain identification and does not estimate event-time accuracy or superiority over published segmentation methods.

The formal participant crosswalk confirmed 10 T0 participants who were not represented in the 108-trial development dataset. In this participant-independent descriptive subset, the frozen adaptive algorithm identified complete ordered P1–P4 chains in 10/10 trials (100.0%; exact 95% CI, 69.2%–100%), with all 10 trials meeting the prespecified QC criteria. Because of the small subgroup size, this analysis was descriptive and was not interpreted as independent validation of event-detection accuracy.

### 3.3. Parameter Sensitivity and Targeted Component Ablation

Across all one-at-a-time perturbations of the 52 numerical parameters to 80%, 90%, 110%, and 120% of their frozen values, complete ordered P1–P4 chain identification remained 108/108 (100%) in the development dataset, 24/24 (100%) in the full T0 batch, and 10/10 (100%) in the participant-independent descriptive subset. No sensitivity-induced chain failure occurred. Boundary placement was less invariant than chain completion. The largest local sensitivities were concentrated in sit-down recovery and P3–P4-related rules; maximum single-trial boundary shifts were approximately 1.0 s in the full analyses and 0.84 s in the participant-independent subset.

Targeted ablation showed that removal of the separate seated-baseline/fallback component reduced complete-chain identification to 101/108 (93.5%) in development, 12/24 (50.0%) in the full T0 batch, and 8/10 (80.0%) in the participant-independent subset. Disabling the unified candidate-chain component or final standing-interval refinement preserved 100% complete-chain identification across all three datasets but produced measurable shifts in selected P2/P3 boundaries. Disabling the final QC mapping did not alter event times or chain completion. These analyses characterize architectural contributions to algorithmic completion and boundary placement rather than event-detection accuracy.

### 3.4. WBB–Noraxon Total-Cycle Comparison

**Figure 5:**
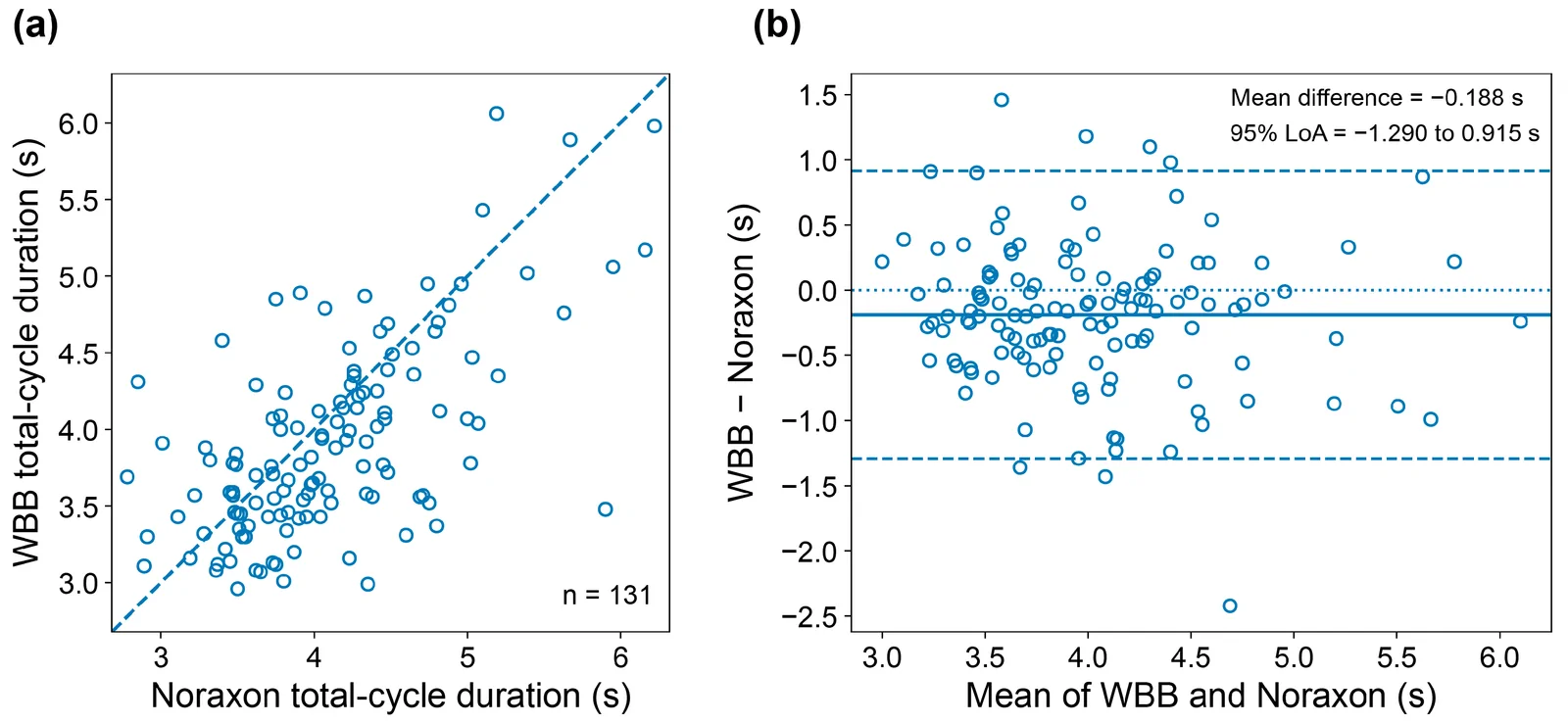
WBB–Noraxon total-cycle duration comparison (*n* = 131 paired trials): (**a**) trial-level WBB-derived versus Noraxon software-reported duration; the dashed line is the line of identity; (**b**) Bland–Altman plot (WBB minus Noraxon): mean difference −0.188 s (solid line), descriptive trial-level 95% limits of agreement −1.290 to 0.915 s (dashed lines), and zero difference (dotted line). The participant-cluster bootstrap 95% CI for the mean difference was −0.313 to −0.060 s. These differences reflect operational timing definitions and are not errors against an independent reference standard.

Across 131 paired trials, mean WBB total-cycle duration was 3.913 ± 0.629 s and mean Noraxon software-reported duration was 4.101 ± 0.678 s. The mean WBB-minus-Noraxon difference was −0.188 s (participant-cluster bootstrap 95% CI, −0.313 to −0.060; participant-cluster-robust *p* = 0.003). Descriptive empirical trial-level limits of agreement were −1.290 to 0.915 s. These differences represent discrepancies between operational timing definitions from different signal domains and should not be interpreted solely as WBB event-detection error or as agreement with an independent reference standard. The corresponding numerical comparison is summarized in Table 2, and the trial-level agreement is illustrated in Figure 5.

Participant-mean ICC(A,1) was 0.572 (bootstrap 95% CI 0.383 to 0.723). No clear proportional bias was detected (slope = −0.093, *p* = 0.346), and the internal-evaluation-minus-development difference in mean bias was 0.034 s (95% CI −0.177 to 0.244; *p* = 0.755).

Values are mean ± SD for 131 paired trials. Differences were calculated as WBB minus Noraxon. Confidence intervals were obtained using participant-cluster bootstrap, and the *p* value was obtained from a participant-cluster-robust model.

### 3.5. Exploratory Synchronized Boundary Analysis

Noraxon report-consistent boundaries were reconstructed for 107 development trials. Split was supported by the C7 signal in all records, whereas the evidence used to define E1 and E4 varied across trials (Supplementary Table S1A,C). Of 93 trials with a seated stomp recorded by both systems, 75 were reliably synchronized and included in the boundary analysis, representing 30 participants.

**Figure 6:**
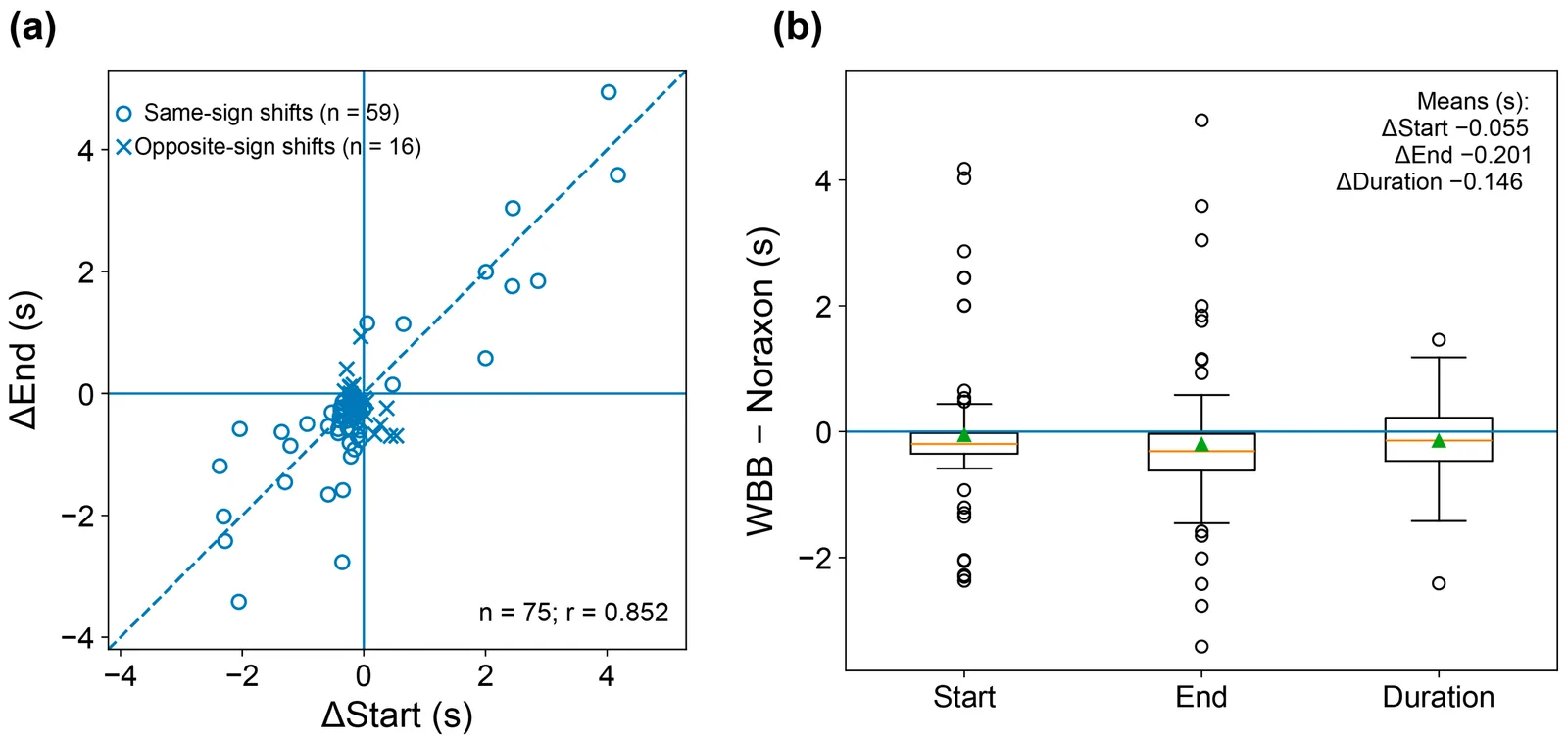
Synchronized WBB–Noraxon boundary differences in 75 trials from 30 participants: (**a**) ΔStart (WBB P1 − Noraxon E1) versus ΔEnd (WBB P4 − Noraxon E4); circles indicate same-sign shifts (*n* = 59), crosses indicate opposite-sign shifts (*n* = 16), and the dashed diagonal line indicates the line of identity. Pearson *r* = 0.852 (participant-cluster bootstrap 95% CI, 0.553 to 0.930); (**b**) distributions of ΔStart, ΔEnd, and ΔDuration; boxes show the interquartile range, center lines medians, whiskers values within 1.5 times the interquartile range, open circles observations beyond the whiskers, and triangles arithmetic means. These operational differences are conditional on successful synchronization, not device-error estimates.

As summarized in Table 3, mean ΔStart was −0.055 s (participant-cluster bootstrap 95% CI −0.408 to 0.369), mean ΔEnd was −0.201 s (95% CI −0.554 to 0.229), and mean ΔDuration was −0.146 s (95% CI −0.322 to 0.020). Their distributions are shown in Figure 6b. The algebraic identity ΔDuration = ΔEnd − ΔStart was satisfied numerically.

ΔStart and ΔEnd were correlated (*r* = 0.852; participant-cluster bootstrap 95% CI 0.553 to 0.930; Figure 6a). This association is descriptive: both quantities share the reconstructed Split framework and the same trial-specific synchronization offset. Thus, the correlation cannot be interpreted as independent evidence of a biological mechanism. In 59/75 trials (78.7%), the two differences had the same sign and partially cancelled algebraically in ΔEnd − ΔStart; in 16/75 (21.3%), opposite signs accumulated. Results restricted to the 25 trials in which both E1 and E4 were signal-supported were directionally similar for ΔDuration (Supplementary Table S2A–C). Inclusion in the synchronized set differed by trial order (global *p* = 0.036 from the generalized estimating equation), but trial-adjusted and inverse probability-weighted sensitivity estimates were similar to the primary estimates. These analyses remain conditional on reliable synchronization and cannot exclude unmeasured selection effects.

Differences were calculated as WBB minus Noraxon. Negative values indicate an earlier WBB boundary or a shorter WBB total-cycle duration. Confidence intervals were obtained using participant-cluster bootstrap. The analysis included 75 reliably synchronized trials from 30 participants.

## 4. Discussion

This study developed an adaptive state- and morphology-based algorithm that derived four principal sit-to-stand-to-sit boundaries from an ordered ten-anchor WBB event chain. Rather than relying on four independent threshold crossings, the algorithm combined movement-interval localization, seated- and standing-load states, peak–trough morphology, complete-chain candidate scoring, and conditional boundary refinement. In the temporally separate T0 internal evaluation batch with participant overlap, the frozen algorithm identified complete P1−P4 chains in all 24 trials, whereas the prespecified internal non-adaptive baseline did so in 7 trials. This result characterizes algorithmic completion under the studied conditions and does not establish event-time accuracy or superiority over published segmentation methods. Repeated trials from the same participant captured trial-to-trial waveform variation and, therefore, contributed distinct waveform patterns to algorithm development, although the full T0 batch was not participant-independent.

The additional robustness analyses clarify what the present algorithmic results do and do not demonstrate. Complete-chain identification remained unchanged across the prespecified ±10% and ±20% one-at-a-time perturbations of 52 numerical parameters in the development dataset, the full T0 batch, and the participant-independent descriptive subset. This reduces concern that complete-chain performance depended on narrowly tuned numerical values. However, selected sit-down recovery parameters produced appreciable P2–P4 boundary shifts in individual trials, demonstrating that robustness of chain completion does not imply invariant boundary timing. Similarly, targeted component ablation showed that the separate seated-baseline/fallback architecture materially contributed to complete-chain output. These findings characterize the behavior of the frozen algorithm under the studied conditions but do not eliminate the possibility of dataset-specific adaptation, establish reference-event accuracy, or demonstrate external generalizability.

The cross-device analyses showed how the same sit-to-stand-to-sit movement was assigned different operational boundaries when derived from WBB loading versus C7 kinematics. The mean WBB-minus-Noraxon difference was small relative to the wide single-trial limits of agreement, which remained nearly unchanged in the repeated-measures sensitivity analysis. The reconstructed Noraxon E1–Split–E4 framework was transparent and report-consistent: Split was signal-supported, whereas some outer boundaries required report-duration constraints or duration-based derivation. This procedure did not reproduce the manufacturer’s event-detection algorithm. Direct force-plate and motion-sensor comparisons have, likewise, shown modality-dependent event timing [21], and combined force and IMU studies indicate that kinetic and kinematic variables describe complementary aspects of chair-rise performance [22,23]. However, ΔStart and ΔEnd shared Split-related reconstruction and a common synchronization offset; ΔDuration was algebraically defined as their difference. Their correlation and partial cancellation, therefore, should not be interpreted as independent mechanistic evidence. The observed pattern is consistent with, but does not prove, modality-dependent placement of cycle boundaries.

Four-event segmentation supplies the temporal structure required for phase-specific analysis of loading rate, center-of-pressure behavior, asymmetry, and postural stabilization. Consistent operational segmentation of a single cycle also provides a foundational building block for automated analysis of repeated chair-rise tests, because each repetition must be identified before counting or phase-level aggregation.

Published STS segmentation methods broadly include transparent rule-based procedures, template or dynamic time-warping approaches, and machine learning or deep learning models [8,24,25,26,27]. Caprioli et al. [28] documented the broader growth of wearable- and IMU-based movement research and the increasing role of machine learning, although that bibliometric analysis was not specific to STS. We selected a deterministic rule-based architecture because its operations are inspectable and operationally interpretable, while remaining reproducible and computationally modest for low-cost WBB signals. The present study did not benchmark these published approaches directly and, therefore, makes no claim of superiority over them.

Low-cost systems nevertheless measure different physical quantities and introduce different technical constraints. The WBB records mechanically coupled vertical-load redistribution without requiring orientation-sensitive body-worn sensor placement; however, its timing depends on foot contact, board calibration, effective sampling frequency, and load-state definitions. In contrast, smartphone video can estimate segment or joint kinematics without an attached inertial unit, whereas accelerometers and gyroscopes embedded in smartphones or smart glasses measure location-dependent linear and angular motion. Their outputs may vary with attachment location, orientation, sampling frequency, filtering, synchronization, integration drift, and device-specific preprocessing. Accordingly, accuracy estimates cannot be directly transferred across WBB, smartphone, smart glasses, and laboratory systems: each approach requires modality-appropriate synchronization and comparison with independent reference events. Wearable and video approaches may offer greater portability, while the present deterministic WBB method offers an inspectable mapping from load morphology to operational boundaries; these are complementary trade-offs rather than evidence that one modality is universally more accurate [7,8,21,28].

The WBB–Noraxon analyses, likewise, compare operational timing definitions rather than a test method against an independent reference standard. WBB boundaries were inferred from mechanical load-state transitions, whereas Noraxon timing was derived from trunk kinematics and software-reported phase durations. Split was signal-supported, while some outer E1/E4 boundaries were report-constrained or duration-derived. The observed Bland–Altman dispersion, therefore, reflects both sensing modality and boundary-definition differences and should not be attributed solely to error in either device.

Several limitations remain. Participants were healthy adults performing a controlled natural-speed task. The temporally separate T0 batch was small and included 14 participants who also appeared in development. Although the remaining 10 trials formed a confirmed participant-independent descriptive subset, that subgroup was too small to constitute an adequately powered independent validation cohort. Successful identification of all four ordered events cannot be interpreted as event-time accuracy because no independent video, optical motion-capture, synchronized laboratory force-platform event labels, or expert sample-level reference annotations were available. The parameter-sensitivity and ablation analyses characterize numerical and architectural robustness but cannot exclude dataset-specific adaptation or establish external clinical generalizability. The internal non-adaptive baseline did not represent all published segmentation approaches. Finally, the synchronized boundary analysis was conditional on successful synchronization, and some Noraxon outer boundaries were report-constrained or duration-derived. Independent, externally sampled, clinically diverse, and reference-annotated studies are, therefore, required before claims regarding event-time accuracy or broader clinical application can be made.

Because inter-foot distance was recorded but not fixed to one standardized value, foot placement may have altered the base of support, trunk strategy, and WBB load-waveform morphology. The present study did not model foot distance as an independent determinant of waveform variability or algorithm performance; future clinical studies should standardize or explicitly model foot position.

## 5. Conclusions

This study developed a frozen adaptive state- and morphology-based algorithm for operational four-event segmentation of WBB vertical-load STS waveforms. Complete ordered P1–P4 chains were identified in 24/24 T0 trials and in the participant-independent descriptive subset (10/10), compared with 7/24 for the prespecified internal non-adaptive baseline. Across ±10% and ±20% one-at-a-time perturbations of 52 frozen numerical parameters, complete-chain identification remained 100% in the 108-trial development set, full T0 batch, and descriptive subset, although selected sit-down rules shifted local boundaries. Removing the separate seated-baseline/fallback component reduced complete-chain identification to 101/108, 12/24, and 8/10. The WBB–Noraxon mean total-cycle difference was −0.188 s (95% CI, −0.313 to −0.060 s; limits of agreement, −1.290 to 0.915 s), reflecting distinct operational timing definitions derived from mechanical-load and kinematic signals rather than error relative to an independent reference standard. Independent reference-event studies and clinically diverse external evaluations remain necessary before claims regarding event-time accuracy or broader clinical generalizability can be made.

## Figures and Tables

**Table 1:** Participant characteristics by acquisition batch.

Variable	Overall Sample (*n* = 46)	Development Batch (*n* = 36)	Internal Evaluation Batch (*n* = 24)
Sex, female/male, *n*	23/23	20/16	9/15
Age, years	26.8 ± 6.2	27.4 ± 6.8	26.3 ± 3.9
Height, m	1.68 ± 0.08	1.68 ± 0.08	1.69 ± 0.09
Body mass, kg	68.2 ± 14.5	67.8 ± 15.1	69.8 ± 11.9
BMI, kg/m^2^	24.1 ± 4.3	23.8 ± 4.3	24.4 ± 3.5
Trials, *n*	132	108	24

**Table 2:** Comparison of total-cycle duration between WBB and Noraxon.

Outcome	WBB	Noraxon	Mean Difference (95% CI)	*p* Value
Total-cycle duration, s	3.913 ± 0.629	4.101 ± 0.678	−0.188 (−0.313 to −0.060)	0.003

**Table 3:** Synchronized boundary differences between WBB and Noraxon.

Boundary Measure	Mean Difference, s (95% CI)
Cycle start, ΔStart	−0.055 (−0.408 to 0.369)
Cycle end, ΔEnd	−0.201 (−0.554 to 0.229)
Total-cycle duration, ΔDuration	−0.146 (−0.322 to 0.020)

## Data Availability

A private version-controlled reproducibility repository contains the WBB reference implementation, algorithm specifications, statistical scripts, deidentified aggregate-analysis table schemas, synthetic waveform data, software-environment files, SHA-256 reproducibility manifests, the complete frozen implementation, and archived final outputs. Access can be provided to editors and reviewers upon request. A versioned public release with a persistent identifier will be archived upon publication. Individual-level raw WBB and Noraxon time series, identifier mappings, and unnecessary demographic source files are not publicly available because unrestricted release is not permitted by participant consent and institutional approval. Additional deidentified analysis materials may be available from the corresponding author for a scientifically justified purpose, subject to data minimization, ethics compatibility, and institutional authorization.

## Supplementary Materials

The following supporting information can be downloaded at: https://www.mdpi.com/article/10.3390/s26196267/s1, Figure S1: Representative deidentified Noraxon C7 waveform illustrating report-consistent boundary reconstruction; Table S1: Noraxon boundary-reconstruction provenance, seated-stomp synchronization flow, and E1-by-E4 boundary-source cross-classification; Note S1: Boundary-provenance definitions and seated-stomp synchronization release logic; Table S2: Boundary-difference sensitivity estimates, selection into the synchronized analysis set, and trials with both E1 and E4 signal-supported; Table S3: Descriptive participant-independent check within the internal evaluation batch; Table S4: WBB preprocessing, movement-interval localization, seated-baseline estimation, state-band formulas, endpoint rules, and final quality-control criteria; Note S2: Ordered execution pathway linking Figure 3 to the detailed algorithm criteria; Table S5: Candidate-chain generation, scoring, ambiguity handling, dominant-standing-bridge refinement, and morphology-specific boundary refinement; Table S6: Frozen prespecified internal non-adaptive baseline (FixedThreshold_D_v1.3_FROZEN) specification; Note S3: Post-review robustness-analysis design; Table S7: Summary of numerical parameter-sensitivity analysis; Table S8: Targeted component-ablation summary; Table S9: Participant-independent descriptive robustness check.
