# Supplementary material for: An Adaptive State- and Morphology-Based Four-Event Segmentation Algorithm for Sit-to-Stand-to-Sit Cycles from Wii Balance Board Load Signals: An Exploratory Comparison with Noraxon Report-Consistent IMU Timing
Source: Sensors (Basel). 2026 Oct 2;26(19):6267. doi: 10.3390/s26196267 (PMC13667418; doi:10.3390/s26196267)
Supplement: Supplementary file 1 [file sensors-26-06267-s001.zip › sensors-4484430-supplementary.pdf]

Supplementary Materials

An Adaptive State- and Morphology-Based Four-Event Segmentation Algorithm for Sit-to-Stand-to-Sit Cycles From Wii Balance Board Load Signals: An Exploratory Comparison With Noraxon Report-Consistent IMU Timing

Supplementary Table S1A. Noraxon boundary-reconstruction provenance.

Component	Result	Interpretation
Valid development Noraxon records	107	One development record lacked a valid same-trial pair
Split supported by C7 signal	107/107	Kinematic phase-separation anchor available in all reconstructed records
E1 boundary source	82 signal-supported; 4 report-constrained; 21 duration-derived	Provenance categories, not independent accuracy grades
E4 boundary source	43 signal-supported; 12 report-constrained; 52 duration-derived	Provenance categories, not independent accuracy grades

Supplementary Table S1B. Seated-stomp synchronization flow.

Flow step	n / result	Interpretation
Planned repeated development records	108	T1–T3 records
Unique WBB-Noraxon pairs	107	One record lacked a valid same-trial pair
Stomp not captured by Noraxon	14	Excluded from synchronization eligibility; not counted as a synchronization failure
Synchronization eligible	93	Unique pair with a captured stomp
Reliably synchronized	75/93 (80.6%)	Exploratory synchronized-boundary set
Not synchronized	18/93 (19.4%)	Excluded without imputation; reasons retained

Supplementary Table S1C. E1-by-E4 boundary-source cross-classification.

E1 source / E4 source	Signal-supported	Report-constrained	Duration-derived	Total
Signal-supported	35	8	39	82
Report-constrained	0	0	4	4
Duration-derived	8	4	9	21
Total	43	12	52	107

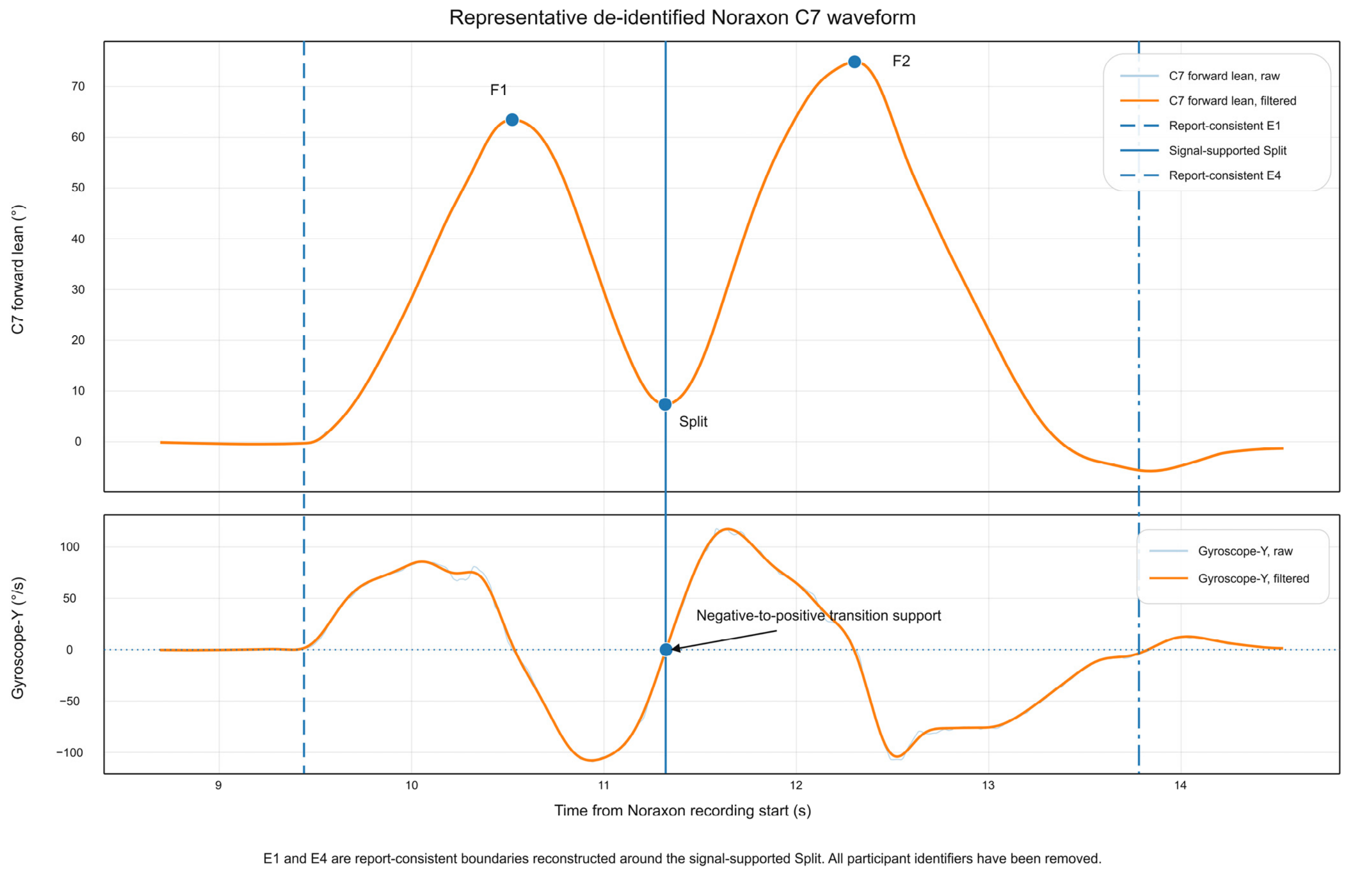

Supplementary Figure S1. Representative deidentified Noraxon C7 waveform illustrating report-consistent boundary reconstruction. F1 and F2 denote the principal forward-lean peaks. Split is the intervening local angle minimum supported by a negative-to-positive zero crossing in the gyroscope Y-axis angular-velocity signal. E1 and E4 were reconstructed relative to Split using the software-reported Stand and Sit durations; they were not sample-level event labels directly exported by Noraxon. Raw and zero-phase-filtered signals are shown.

Supplementary Note S1. Boundary-provenance definitions and seated-stomp synchronization release logic.

Boundary provenance. Signal-supported indicates that an outer signal-derived candidate satisfied the corresponding signal rule and the report-duration consistency check. Report-constrained indicates that a signal candidate was retained only after applying the same-trial software-reported phase duration as a constraint. Duration-derived indicates that no usable outer signal candidate was available but Split was reliable, so the boundary was calculated directly as E1 = Split - Stand Duration or E4 = Split + Sit Duration. These categories describe evidential provenance and are not independent accuracy grades; the implementation-level tolerances are retained in the frozen reviewer code.

Seated-stomp synchronization. Within each eligible trial, WBB impact candidates and Noraxon right-thigh kinematic impact candidates were ranked and jointly paired. The formal offset used impact onset (WBB onset minus Noraxon onset); the peak-based offset and onset-peak disagreement were quality-control measures only. An official synchronization offset was assigned only when a unique best pair passed the frozen morphology, candidate-separation, side-orientation, contralateral-thigh immobility, and cross-system pairing checks. Trials classified for review retained provisional candidates but were not assigned an official offset and were excluded from boundary analysis. Among the 18 reviewed trials, the primary reasons were insufficient active-side unloading dominance (n = 6), excessively broad WBB impact (n = 4), ambiguous joint pairing (n = 3), excessive contralateral-thigh activity (n = 2), absence of clear return to seated load after impact (n = 2), and insufficient active-side landing dominance (n = 1). No offset was imputed.

Supplementary Table S2A. Boundary-difference sensitivity estimates.

Outcome	Primary estimate	Trial-adjusted estimate	IPW estimate
ΔStart, s	-0.055 (-0.408 to 0.369)	-0.040 (-0.448 to 0.367)	-0.040 (-0.443 to 0.362)
ΔEnd, s	-0.201 (-0.554 to 0.229)	-0.195 (-0.609 to 0.220)	-0.195 (-0.604 to 0.214)
ΔDuration, s	-0.146 (-0.322 to 0.020)	-0.154 (-0.329 to 0.021)	-0.154 (-0.326 to 0.017)

Supplementary Table S2B. Selection into the synchronized analysis set.

Assessment	Result
Eligible / synchronized / not synchronized	93 / 75 / 18

T1 inclusion	25/31 (80.6%)
T2 inclusion	22/31 (71.0%)
T3 inclusion	28/31 (90.3%)
GEE global trial effect	$p = 0.036$
T3 vs T2 inclusion	OR 3.82 (95% CI, 1.35–10.76); Holm $p = 0.034$
Maximum absolute demographic SMD	0.182
IPW range / effective sample size	0.893–1.136 / 74.3 of 75
Observed cycle-variable models	No statistically significant predictor after trial adjustment

Supplementary Table S2C. Trials with both E1 and E4 signal-supported.

Outcome	Result / interpretation
Trials / participants	25 / 18; subset of the 75 reliably synchronized trials
ΔStart, s	-0.292 (-0.514 to -0.130)
ΔEnd, s	-0.446 (-0.794 to -0.189)
ΔDuration, s	-0.154 (-0.404 to 0.011); directionally similar to the full synchronized set
ΔStart–ΔEnd correlation	$r = 0.686$; descriptive because shared reconstruction and synchronization components remain
Same-sign start and end differences	22/25 (88.0%); algebraic partial cancellation in ΔEnd - ΔStart

Primary confidence intervals are participant-cluster bootstrap intervals. Trial-adjusted estimates used cluster-robust models, and stabilized inverse-probability weights included trial order only. Demographic balance was summarized using standardized mean differences for the recorded age, sex, height, body mass, and BMI variables. Cycle-variable models used the observed WBB and Noraxon cycle-duration measures available in the aligned-eligibility dataset. These analyses cannot exclude unmeasured selection.

Supplementary Table S3. Descriptive participant-independent check within the internal evaluation batch.

Item	Result / interpretation
Participants / trials	10 participants / 10 single internal-evaluation trials
Relationship to development batch	Participants were not represented in the 108-trial development batch
Adaptive complete P1–P4 identification	10/10 (100.0%; exact 95% CI, 69.2% to 100.0%); all four ordered events were identified in every trial
Review or Exclude outcomes	0/10
Interpretation	Descriptive participant-independent check; the subgroup was too small for independent validation

This subset analysis is descriptive because the subgroup was small and was too small to support independent validation.

Supplementary Table S4. WBB preprocessing, movement-interval localization, seated-baseline estimation, state-band formulas, endpoint rules, and final quality-control criteria.

Decision step	Finalized algorithm criterion	Purpose
Principal movement-interval localization	Let $R = \max[Q0.95(Ws) - Q0.10(Ws), 1.0\ \text{kg}]$ and $Tactivity = Q0.10(Ws) + 0.35R$. Fill above-threshold gaps ≤0.25 s, retain candidate segments lasting ≥1.0 s, and select the segment with the largest positive area above Tactivity. Extend the search region 2.0 s before and 3.0 s after the selected segment.	Localize the main sit-to-stand-to-sit excursion within the full recording before peak, trough, baseline, and boundary searches.
Signal preprocessing	After invalid observations were removed and duplicate time stamps were averaged, $\Delta t\_eff = \text{median}\{t\_i - t\_(i-1): t\_i > t\_(i-1)\}$ and $f\_eff = 1/\Delta t\_eff$. A duration a was converted as $N(a) = \max[n\_min, \text{round}(a\ f\_eff)]$, with a module-specific n_min; for centered filters, one sample was added when N(a) was even. A centered 0.05-s rolling median was followed by a centered 0.075-s moving mean.	Define the actual timestamp-derived sampling rate and the two-stage preprocessing used by the finalized algorithm.

Decision step	Finalized algorithm criterion	Purpose
Initial unified-SBL candidates	For each 0.75-s window B searched at an approximately 0.15-s step, compute μ_B, σ_B, range R_B, and OLS slope β_B. Stable if $\sigma\_B \le \max[0.50 \text{ kg}, 0.04 \times \max(\lvert\mu\_B\rvert, 1.0 \text{ kg})]$ and $\lvert\beta\_B\rvert \le 1.5$ kg/s. Let $A = \max[Q\_0.95(W) - Q\_0.10(W), 1.0 \text{ kg}]$ and $q\_0.35 = Q\_0.35(\{\mu\_B\text{: B stable}\})$; retain low-load windows satisfying $\mu\_B \le q\_0.35 + 0.05A$. Range contributes to ranking but is not a separate exclusion threshold.	Identify stable low-load candidates using the finalized selection rule.
Unified Pre/Post-SBL compatibility	For best pre- and post-sitting candidates, declare compatibility when $\lvert\mu\_pre - \mu\_post\rvert \le \max\{2 \times \max(\sigma\_pre, \sigma\_post), 0.08 \times \max(\lvert\mu\_pre\rvert, 1.0 \text{ kg})\}$. If compatible, $\mu\_SBL = (\mu\_pre + \mu\_post)/2$ and $\sigma\_SBL = \max(\sigma\_pre, \sigma\_post)$. If not, the difference is recorded; mismatch alone is insufficient to invoke separate-baseline estimation.	Define when one seated-load state can represent both sides of the movement.
Candidate durations for conditional separate-baseline estimation	3.0, 2.5, 2.0, 1.5, 1.0, 0.75, and 0.5 s; 0.10-s search step.	Identify stable local Pre-SBL and Post-SBL windows when the prespecified conditions are met.
Low-load criterion for conditional separate-baseline estimation	Window mean < standing mean − 0.30 × max(standing mean − signal 10th percentile, 1.0 kg).	Restrict candidates to values below the standing-load state.
Stability criteria for conditional separate-baseline estimation	SD ≤ max[0.30 kg, 0.03 × max(|mean|, 1.0 kg)]; range ≤ max[0.75 kg, 0.06 × max(|mean|, 1.0 kg)]; |slope| ≤ 1.2 kg/s.	Exclude noisy or drifting seated windows.
Baseline estimate	For the selected window B: $\mu\_SBL = \text{mean}(W\_t \mid t \in B)$; $\sigma\_SBL = \text{SD}(W\_t \mid t \in B)$.	Define the local seated-load centre and variability.
Seated-state band	$\delta\_SBL = \max[2\sigma\_SBL, 0.025 \times \max(\lvert\mu\_SBL\rvert, 1.0 \text{ kg})]$; $L\_SBL = \mu\_SBL - \delta\_SBL$; $U\_SBL = \mu\_SBL + \delta\_SBL$.	Convert the selected baseline into the load-state bounds used by P1 and standard endpoint decisions.
Conditional Pre-SBL search	Up to 10 s before PrSUT (or the pre-rise boundary when PrSUT is unavailable); select the candidate using distance and stability scoring.	Estimate Pre-SBL for P1 when separate-baseline conditions are met.
Conditional Post-SBL search	0.10–8.0 s after PST; the following 1.50 s must not show an excessive mean shift, range, or return toward standing load.	Estimate Post-SBL for P4 when separate-baseline conditions are met. The 8.0-s value is a post-sitting search limit, not a peak-search window.
P1 initial candidate	First run below L_SBL lasting ≥0.05 s within the sequence leading to the final PrSUT–SUP rise.	Identify sustained departure from the seated-load state rather than the end of the baseline window.
P1 false-start rejection and refinement	If an early below-band run is followed by re-entry into [L_SBL, U_SBL] for ≥0.15 s before the main rise, search restarts after the final stable seated segment. The candidate is then refined against the final PrSUT–SUP sequence and accepted only within the ordered event sequence.	Exclude preparatory unloading or false-start-like fluctuations from the final cycle start.
Conditions for separate-baseline estimation	Missing P1 or P4; endpoint failure P1_not_visible, PrSUT_not_visible, or P4_not_visible; P1-to-PrSUT < 0.08 s; P1-to-SUP > 2.0 s; PST-to-P4 < 0.20 s; or the P4-specific combination PST-to-P4 = 0.85–1.25 s, Post-SBL start ≤0.70 s after PST, Post-SBL at least 0.50 kg below the unified baseline, and a high-risk quality-control classification.	Restrict separate-baseline estimation to explicit endpoint or waveform-sequence conditions rather than baseline mismatch alone.
Baseline mismatch	Record the Pre-Post mean difference in kg and percent; mismatch alone is insufficient to invoke separate-baseline estimation.	Describe baseline behavior without post hoc switching.
P4 confirmation	The first stable entry uses a 0.30-s window and is rejected if a subsequent large dip or rise occurs before stable post-sitting behavior is established.	Avoid assigning P4 before a stable post-sitting state is reached.
Relaxed Post-SBL range for conditional P4 estimation	Post-SBL mean ± 3 × Post-SBL SD, combined with the 0.30-s SD, range, slope, target-distance, and future-stability criteria.	This ±3-SD range is confined to conditional P4 estimation with separate baselines; it is not the general seated-state band or P1 threshold.

Decision step	Finalized algorithm criterion	Purpose
Final quality-control classification	OK required all internal anchors to be present and ordered as P1 < PrSUT < SUP < PSUT < P2 ≤ P3 < PSDT < SDP < PST < P4; total-cycle duration 2–8 s; P1-to-SUP ≥0.25 s; and no unresolved endpoint or morphology warning. PST-to-P4 <0.20 s remained a review condition unless the finalized early stable-platform criterion was satisfied. Unresolved trials were classified as Review or Exclude.	Define complete event identification consistently without manual event-time correction.

Abbreviations and symbols: W_t, WBB vertical load at time t; B, selected candidate window; SBL, seated baseline/load state; PrSUT, pre-standing-up trough; SUP, standing-up peak; PSUT, post-standing-up trough; PSDT, pre-sitting-down trough; SDP, sitting-down peak; PST, post-sitting trough; Q_p, p-th quantile; μ, mean; σ, standard deviation; β, ordinary least-squares slope; OLS, ordinary least squares.

All formulas and thresholds in Tables S4 and S5 were extracted directly from the frozen final source code. They were specified using T1–T3 and applied without modification to the formal T0 internal evaluation. These values are operational definitions for this algorithm rather than universal biomechanical standards. Their functional roles are reported, but available records do not support claims of grid-search optimization or parameter optimality. Source-file hashes, allowed release transformations, synthetic parity, and aggregate restricted-data parity are retained in the reviewer reproducibility repository.

Supplementary Note S2. Ordered execution pathway linking Figure 3 to the detailed algorithm criteria.

Figure 3 summarizes the control flow of the adaptive state- and morphology-based ordered-event-chain algorithm rather than every numerical condition. The procedure operates in the following order: validate and preprocess the time-stamped load signal; localize the principal movement interval; estimate the initial unified seated-load state and core morphology anchors; construct the initial ordered ten-anchor chain; invoke independent-baseline fallback or another recovery module only under explicit endpoint or sequence triggers; generate complete candidate chains and apply hard constraints; score candidates and assess ambiguity; refine P2/P3 against the dominant standing-load bridge and P1/P3/P4 under morphology-specific triggers; and perform final quality control. Table S4 lists preprocessing, movement-interval, baseline, endpoint, and final quality-control rules; Table S5 lists candidate generation, scoring, ambiguity handling, and selected-chain boundary-refinement rules. No step uses Noraxon data, participant identity, a manually specified trial-specific temporal landmark, or manual event-time correction.

Supplementary Table S5. Candidate-chain generation, scoring, ambiguity handling, dominant-standing-bridge refinement, and morphology-specific boundary refinement.

Decision component	Finalized criterion	Role in the ordered execution pathway
Candidate-chain set	The standard chain was supplemented, when activated, by candidates based on an independently recovered standing episode, joint SUP/PSUT relocation, phase-aware sitting-down-chain recovery, and P2/P3 reassessment after sitting-chain recovery. A recovery module that was not activated produced no valid candidate.	Provide explicit alternatives for prespecified atypical morphology without changing global rules at run time.
Hard constraints before scoring	Every valid candidate had to satisfy P1 < PrSUT < SUP < PSUT < P2 ≤ P3 < PSDT < SDP < PST < P4, positive sit-to-stand and stand-to-sit transition durations, positive total-cycle duration, and no existing Exclude status.	Remove incomplete or temporally impossible chains before comparative scoring.
Candidate-specific standing reference	Thigh = max[Q0.90(Ws), muSBL + 0.60(max(Ws) - muSBL)]. The median and SD of samples at or above Thigh defined the initial high-load reference; Q0.85 was used when fewer than three samples were available. Candidate P2-P3 intervals were then evaluated using robust MAD-, SD-, and relative-tolerance bounds.	Allow the standing-load state to be evaluated relative to the candidate chain rather than a single fixed absolute threshold.
Six-domain candidate score	Schain = clip[0,1](0.12Speak + 0.28Sstand + 0.18SP2 + 0.16SP3 + 0.14Send + 0.12Squality - sum Penalty). The domains represented peak-trough structure, standing-episode quality, P2 entry, P3 exit, endpoint/duration plausibility, and existing quality evidence.	Rank complete chains using morphology, state occupancy, temporal plausibility, and signal-quality evidence.

Decision component	Finalized criterion	Role in the ordered execution pathway
Suspicion score and penalties	Ten weighted suspicion components covered P2 proximity to PSUT, P2 below the standing core, rapid P2 recovery, P3 at a local peak, P3 proximity to PSDT, confirmed standing re-entry, episode fragmentation, a short high-load island, abnormally short standing, and chain ambiguity. Standing duration >4.0 s incurred 0.10 penalty; suspicion ≥0.55 incurred 0.08; each major boundary red flag ≥0.80 incurred 0.05, capped at 0.12.	Reduce support for chains with unstable or internally contradictory boundary evidence.
Candidate selection and unresolved ambiguity	An alternative chain replaced the standard chain when its final score advantage exceeded 0.08. When the advantage was ≤0.08 and changes in SUP, PSUT, P2, and P3 were all ≤0.05 s, the standard chain was retained as materially equivalent. A top-two score difference <0.08 without the equivalence protection was flagged as unresolved ambiguity and forced Review.	Prevent trivial switching while avoiding manual resolution of genuinely competing chains.
Dominant standing-load bridge	Between PSUT and PSDT, samples at or above the local Q0.55 load were grouped after internal gaps ≤0.22 s were merged. Candidate bridge score was 0.42 times duration coverage + 0.34 times entry-bound occupancy + 0.24 times continuity. The highest-scoring segment was selected; a single-point high-load fallback could form a low-support short bridge.	Define the high-load episode against which P2 entry and P3 exit are refined.
P2 refinement	Search from PSUT to 0.12 s before the dominant-bridge start. A 0.14-s confirmation window (minimum four samples) required recovery from PSUT by at least max[1.5 kg, 0.035 × |standing mean|], future holding-bound occupancy ≥0.65, acceptable early mean derivative, and future stability. The first point satisfying all conditions was selected.	Place P2 at sustained entry into the dominant standing-load bridge rather than at a single crossing.
P3 refinement	A 0.16-s confirmation window (minimum five samples) required negative-derivative occupancy ≥0.60, net load decrease ≥max[0.8 kg, 0.012 × |standing mean|], and no subsequent return to the standing-bridge entry bound before PSDT. A local-peak candidate was moved to the true onset of sustained decline.	Place P3 at final sustained departure from standing and prevent premature assignment followed by re-entry.
P2 = P3 and standing re-entry	P2 = P3 was allowed only when the original interval was no more than one effective sample, P2-entry and P3-exit scores were each ≥0.45, total chain score was ≥0.55, and no unresolved top-candidate ambiguity remained. Post-P3 standing re-entry was confirmed by a 0.15-s window with standing-core occupancy ≥0.65, effective duration ≥0.12 s, and stability score ≥0.55; confirmed re-entry required P3 reset and Review.	Represent a genuinely nonseparable standing interval while rejecting a premature P3 followed by renewed standing.
P4 morphology-specific refinement	Attempted only when current PST-to-P4 was ≥0.55 s. The earliest 0.24-s low-load platform had to satisfy adaptive mean, SD, range, and slope limits and show no marked rise or drift over the following 0.65 s. The replacement had to remain after PST, precede the old P4, and advance the boundary by at least 0.35 s.	Move P4 to the earliest defensible stable post-sitting platform without accepting a transient crossing.
P1 morphology-specific refinement	Attempted only when current P1-to-SUP was ≥0.95 s. The method searched for the final adaptive low-load trough before PrSUT, with a minimum 0.20-s forward shift from the old P1; staged fallbacks examined the pre-PrSUT low point and the preload trough before sustained rapid rise while restricting the preceding signal range.	Exclude prolonged preparatory unloading and place P1 at the final low-load entry into the principal standing-up sequence.

Decision component	Finalized criterion	Role in the ordered execution pathway
P3 early-decline refinement	Attempted only when PSDT - P3 ≤0.12 s. From 0.15 s after P2, a 0.14-s future window required at least 45% of derivatives below the adaptive negative threshold and load decrease ≥max[1.0 kg, 0.018 × standing-seated scale]. The new P3 had to be at least 0.15 s earlier and preserve the complete event order.	Recover the onset of sustained sitting-down decline when the initial P3 was placed too close to PSDT.

Abbreviations: PrSUT, pre-standing-up trough; SUP, standing-up peak; PSUT, post-standing-up trough; PSDT, pre-sitting-down trough; SDP, sitting-down peak; PST, post-sitting trough; SBL, seated-load baseline/state; MAD, median absolute deviation; Q_p, p-th quantile of the smoothed load signal W_s. The score and refinement settings are development-derived engineering parameters fixed before formal T0 evaluation; they are not universal biomechanical thresholds and were not established by grid-search optimization.

Supplementary Table S6. Frozen prespecified internal non-adaptive baseline (FixedThreshold_D_v1.3_FROZEN) specification.

The prespecified internal non-adaptive baseline used parameter set FixedThreshold_D_v1.3_FROZEN. Its parameters were selected using the T1–T3 development records and were frozen before formal T0 processing. No recovery module, trial-specific hard coding, external-device information, or manual event-time correction was used; unsuccessful trials remained in the denominator. A_d denotes dynamic amplitude; Pre-SBL and Post-SBL denote the pre- and post-sitting seated-load baselines; StBL denotes the standing-load reference; W_s denotes smoothed WBB vertical load; and Q0.99 denotes the 99th percentile.

Decision component	Frozen v1.3 rule	Purpose
Version and analytic role	FixedThreshold_D_v1.3_FROZEN; development on T1–T3 only; unchanged application to T0; no post-validation parameter changes.	Defines the frozen deterministic comparator used for the primary internal-evaluation comparison.
Analysis interval and stomp exclusion	When a seated stomp was available, the search began 0.50 s after the stomp and used the first eligible stable pre-sitting window; otherwise it began at the start of the record. Minimum analysis duration was 5.0 s.	Exclude the synchronization stomp from event detection and require an adequate analysis interval.
Signal preprocessing	Centered zero-phase 0.05-s moving-average smoothing; time windows were converted using the trial-specific median sample interval.	Apply the fixed comparator's sole smoothing operation.
Pre- and post-sitting baselines	Search for a 1.0-s stable window within the first and last 3.0 s, respectively. Stability required CV ≤ 0.025, absolute slope ≤ 0.50 kg/s, and range ≤ 0.05 times the window median.	Define the fixed seated-load references without adaptive fallback.
Dynamic amplitude	A_d = Q0.99(W_s) - median(Pre-SBL). Valid only when A_d > max[5.0 kg, 0.10 times median(Pre-SBL)].	Reject records without a sufficiently large load excursion.
Principal peaks	Peak height ≥ median(Pre-SBL) + 0.70A_d; prominence ≥ 0.10A_d; minimum peak separation 0.50 s. The first and last qualifying peaks were SUP and SDP.	Identify the two principal load peaks by fixed criteria.
Standing-load reference	StBL was the 80th percentile between SUP + 0.20 s and SDP - 0.20 s when the interior interval was at least 0.30 s; otherwise the central 50% of the SUP–SDP interval was used. StBL had to exceed median(Pre-SBL) + 0.30A_d.	Define a fixed standing-load level and reject an invalid standing reference.
P1 detection and false-start rejection	Within 3.0 s before SUP, P1 was the final downward crossing below 0.975 times median(Pre-SBL) that remained below for 0.10 s with ≥ 0.80 occupancy. A candidate was rejected if the signal subsequently returned for 0.20 s with ≥ 0.80 occupancy to [0.975, 1.025] times the pre-sitting median before SUP.	Assign sustained cycle onset while rejecting a return to the seated baseline.
P2 detection	First point after SUP within [0.975, 1.025] times StBL for 0.10 s with ≥ 0.80 occupancy.	Assign fixed entry into the standing-load band.
P3 detection	First downward crossing below 0.985 times StBL after P2 that remained below for 0.10 s with ≥ 0.80 occupancy. Intervals shorter than 0.10 s were merged to P2 and then evaluated by the final strict event-order rule.	Assign fixed departure from standing and retain the comparator's prespecified merge handling.
P4 detection	First point after SDP within [0.975, 1.025] times median(Post-SBL) for 0.20 s with ≥ 0.80 occupancy.	Assign stable return to the post-sitting baseline.
Internal trough anchors	PrSUT, PSUT, PSDT, and PST were the minimum smoothed-load samples within P1–SUP, SUP–P2, P3–SDP, and SDP–P4, respectively.	Complete the fixed ten-anchor chain used for final ordering checks.
Final event order	P1 < PrSUT < SUP < PSUT < P2 < P3 < PSDT < SDP < PST < P4.	Reject incomplete or non-strictly ordered fixed-threshold chains.
Total-cycle plausibility	P4 - P1 had to be between 1.5 and 10.0 s inclusive of the stated limits.	Reject implausible fixed-threshold total durations.
Failure handling	Invalid sampling, unstable seated baseline, insufficient amplitude, fewer than two peaks, invalid StBL, a missing event, event-order failure, or implausible duration produced Exclude_fixed; no boundary was manually completed.	Keep all unsuccessful comparator trials in the primary denominator.

Supplementary Note S3. Post-review robustness-analysis design

The parameter-sensitivity and component-ablation analyses were added in response to peer review. The sensitivity-analysis parameter list, perturbation grid, and outcome definitions were frozen before sensitivity outcomes were generated. Fifty-two numerical parameters from the frozen Adaptive v9 implementation were perturbed one at a time to 80%, 90%, 100%, 110%, and 120% of their frozen values, with all other settings unchanged. Analyses were performed in the 108-trial development dataset, the full 24-trial T0 batch, and the confirmed 10-trial participant-independent descriptive subset. Outcomes were complete ordered P1–P4 chain identification, P1–P4 absolute boundary shifts relative to the frozen nominal implementation, total-cycle-duration shifts, and failure topology. Targeted component ablations were performed without re-optimizing remaining parameters. These analyses characterize algorithmic completion and boundary stability rather than event-detection accuracy.

Supplementary Table S7. Summary of numerical parameter-sensitivity analysis

No sensitivity-induced chain failure occurred. The largest development shift was associated with sitdown_recovery_minimum_p3_drop_fraction at factor 0.80, and the largest full-T0 shift was associated with sitdown_recovery_minimum_p3_drop_gain_fraction at factor 1.10. Complete-chain robustness therefore did not imply invariant boundary placement.

Dataset	Frozen nominal completion	Perturbation grid	Completion across all tested perturbations	Largest observed boundary shift
Development	108/108 (100%)	0.80×, 0.90×, 1.00×, 1.10×, 1.20×; 52 OAT parameters	100% complete-chain identification at every tested level	P3: 1.030 s maximum; p95 0.000 s (sitdown_recovery_minimum_p3_drop_fraction, factor 0.80)
Full T0	24/24 (100%)	Same frozen grid	100% complete-chain identification at every tested level	P3: 1.020 s maximum; p95 0.272 s (sitdown_recovery_minimum_p3_drop_gain_fraction, factor 1.10)
Participant-independent T0	10/10 (100%)	Same frozen grid	100% complete-chain identification at every tested level	P3: 0.840 s maximum; p95 0.462 s (sitdown_recovery_minimum_rise_fraction and sitdown_recovery_p3_psdt_near_s)

Supplementary Table S8. Targeted component-ablation summary

The ablation results are interpreted as architectural contribution analyses. They do not establish event-detection accuracy. The pronounced loss of complete-chain output after disabling the separate seated-baseline/fallback component indicates a material contribution of that component to completion robustness, particularly in the full T0 batch.

Ablation condition (frozen module identifier)	Development	Full T0	Participant-independent T0
Full frozen Adaptive v9 reference (full_frozen_reference)	108/108 (100%)	24/24 (100%)	10/10 (100%)
Separate seated-baseline/fallback disabled (disable_separate_sbl_fallback)	101/108 (93.5%)	12/24 (50.0%)	8/10 (80.0%)
Unified candidate chain disabled (disable_unified_candidate_chain)	108/108 (100%)	24/24 (100%)	10/10 (100%); selected P3 shifts
Final standing-interval refinement disabled (disable_final_standing_interval_refinement)	108/108 (100%)	24/24 (100%)	10/10 (100%); selected P2/P3 shifts
Final QC mapping disabled (disable_final_review_rule_qc_mapping)	108/108 (100%)	24/24 (100%)	10/10 (100%); no event-time change

Supplementary Table S9. Participant-independent descriptive robustness check

Item	Result / interpretation
Participants / trials	10 participants / 10 single T0 trials; confirmed absent from the 108-trial development dataset
Frozen Adaptive v9	10/10 complete ordered P1–P4 chains (100.0%; exact 95% CI, 69.2%–100%); 10/10 QC=OK
Sensitivity	100% complete-chain identification throughout all 52-parameter OAT perturbations at ±10% and ±20%; no chain failure
Targeted ablation	Separate seated-baseline/fallback removal: 8/10 complete; other listed ablations: 10/10 complete with selected boundary shifts
Interpretation	Participant-independent descriptive robustness check; not an adequately powered independent validation and not evidence of event-time accuracy
